# Fabrication of Highly Porous and Pure Zinc Oxide Films Using Modified DC Magnetron Sputtering and Post-Oxidation

**DOI:** 10.3390/ma14206112

**Published:** 2021-10-15

**Authors:** Se-Yong Park, Soon-Ho Rho, Hwan-Seok Lee, Kyoung-Min Kim, Hee-Chul Lee

**Affiliations:** Department of Advanced Materials Engineering, Korea Polytechnic University, Siheung 15073, Korea; gastel88@gmail.com (S.-Y.P.); gocnd1238@naver.com (S.-H.R.); ghkstjr7518@gmail.com (H.-S.L.); kkm386@kpu.ac.kr (K.-M.K.)

**Keywords:** zinc oxide, porous film, post-oxidation, DC magnetron sputtering, oxidation mechanism

## Abstract

Porous films of metals and metal oxides exhibit larger surface areas and higher reactivities than those of dense films. Therefore, they have gained growing attention as potential materials for use in various applications. This study reports the use of a modified direct current magnetron sputtering method to form porous Zn-ZnO composite films, wherein a subsequent wet post-oxidation process is employed to fabricate pure porous ZnO films. The porous Zn-ZnO composite films were initially formed in clusters, and evaluation of their resulting properties allowed the optimal conditions to be determined. An oxygen ratio of 0.3% in the argon gas flow resulted in the best porosity, while a process pressure of 14 mTorr was optimal. Following deposition, porous ZnO films were obtained through rapid thermal annealing in the presence of water vapor, and the properties and porosities of the obtained films were analyzed. An oxidation temperature of 500 °C was optimal, with an oxidation time of 5 min giving a pure ZnO film with 26% porosity. Due to the fact that the films produced using this method are highly reliable, they could be employed in applications that require large specific surface areas, such as sensors, supercapacitors, and batteries.

## 1. Introduction

Unlike conventional films, porous films contain pores between their component particles and can be fabricated using both metals and metal oxides. Due to the fact that porous films exhibit larger surface areas and higher reactivities than those of dense films, they have gained growing attention as potential materials for use in various fields, such as energy storage and conversion, catalysis, and sensors [1,2,3,4,5,6,7,8,9].

For example, porous zinc oxide films are known to possess excellent semiconductor properties with wide band gaps (i.e., ~3.37 eV), thereby rendering them suitable for application in gas sensors. More specifically, metal oxide semiconductor-based gas sensors are particularly desirable due to their good stability, superior selectivity, and fast reaction/recovery rates [10,11,12]. Porous zinc oxide films are also n-type semiconductor materials and, thus, are suitable for use in solar cells [13], photocatalysts [14], varistors, and electrodes. They are relatively inexpensive and are easy to fabricate in various forms, such as nanorods, nanoflakes, and nanofibers [15,16,17].

The ZnO nanoparticles used for the preparation of pure porous ZnO films are commonly obtained through wet methods, such as hydrothermal, ball milling, sol–gel, and co-precipitation methods [18,19,20,21]. Among them, the sol–gel method is most commonly used because of its simplicity, despite the fact that it is environmentally less desirable.

Alternatively, dry fabrication methods can also be considered, including the gas evaporation/condensation, aerosol [22], and sputtering routes [23]. However, the first two methods are not applicable for metals with high melting points and are particularly costly, while the aerosol method involves a higher safety risk and greater maintenance costs due to the high pressures required during its operation. In contrast, the sputtering method is eco-friendly compared to the above-mentioned wet methods and allows a high degree of freedom regarding the deposition materials and substrates employed. It was therefore selected for use in the current study to fabricate pure porous ZnO films.

Compared with the vapor–liquid–solid process, the vapor–solid process, the metal–organic chemical vapor deposition route [24,25,26,27], and vapor phase methods such as solution formation [28,29,30], the wet post-oxidation method is advantageous for the preparation of nanostructures via oxidation routes since it allows efficient process control and reduces pollution through lower energy consumption. In addition, the nanostructures formed using this method tend to exhibit a high plasticity and excellent adhesion to the corresponding substrate [31]. However, a number of studies have demonstrated that the mass production of ZnO nanostructures is difficult under a dry oxygen atmosphere, with water vapor playing an important role in the oxidation process to produce the desired nanostructures [32,33].

Thus, we herein report the use of a modified direct current (DC) magnetron sputtering method to form porous Zn-ZnO composite films, wherein a subsequent wet post-oxidation process is employed to fabricate pure porous ZnO films. More specifically, following the formation of porous Zn-ZnO composite films in clusters using various pressures and O_2_ ratios, the deposition thicknesses, particle sizes, and porosities of the obtained films are measured. Subsequently, porous ZnO films are fabricated through rapid thermal annealing (RTA) in a water vapor environment, and the changes in the properties and porosities as a function of the heat treatment time and temperature are analyzed. Finally, the oxidation mechanism responsible for the formation of these pure porous ZnO films is examined.

## 2. Materials and Methods

**Figure 1:**
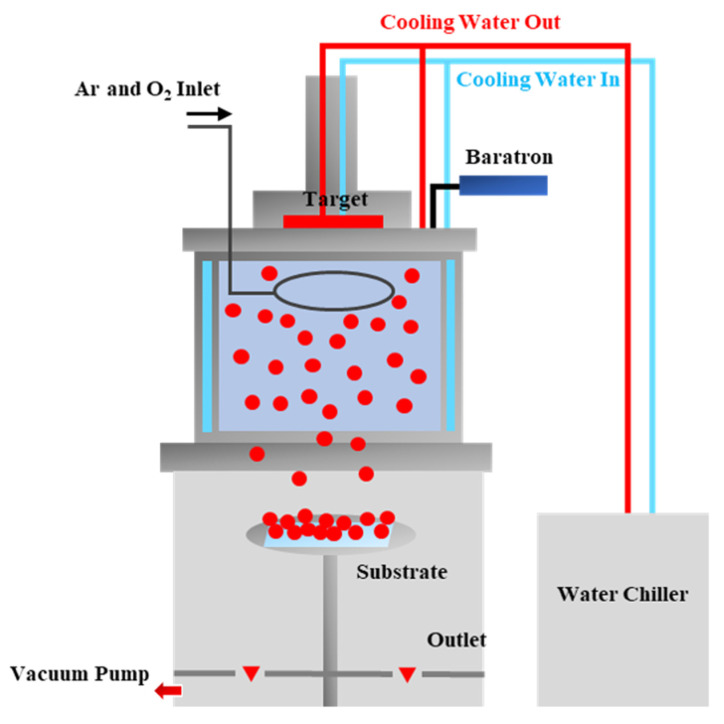
Schematic diagram of the modified DC magnetron sputtering system used in this study [34].

Figure 1 shows a schematic diagram of the modified DC magnetron sputtering system used to form the porous Zn-ZnO composite films, wherein the system is divided into two independent upper and lower parts. As indicated, using this process, plasma is formed in the upper chamber and Zn particles are sputtered at the target, which is located in the lower chamber. As the sputtered zinc particles lose energy through contact with the cooling system on the upper chamber wall and diffuse into the lower chamber, they collide with one another to form clusters of various sizes. During this process, a small amount of O_2_ is introduced to oxidize the Zn particles and form composite films containing Zn and ZnO. For the purpose of this study, a zinc target with a purity of 99.99% was used, and the distance between the target and the substrate was 18 cm. In addition, the flow rate of Ar gas was 10 sccm, while O_2_ gas was injected at a ratio of 0–0.7% relative to Ar. The experiment was performed at a process pressure range of 7–20 mTorr, and the substrates were deposited for 60 min at 150 W. The temperature of the chiller was maintained at 20 °C. Dry-oxidized Si wafers with a thickness of 2000 Å were used as the deposition substrates.

The deposited porous Zn-ZnO composite films were subjected to wet post-oxidation using an RTA instrument under a water vapor atmosphere to form the pure porous ZnO films. The experiment was conducted at a range of temperatures (i.e., 400–600 °C, N.B. melting point of Zn = 420 °C) and reaction times (i.e., 5–30 min). N_2_ gas was used as the carrier gas and was introduced through a bubbler at a flow rate of 0.7 L/min to supply water vapor into the chamber for oxidation. The conditions used for the deposition and heat treatment process are summarized in Table 1.

The geometric shapes and deposition rates of the fabricated porous Zn-ZnO composite films and the pure porous ZnO films were observed using surface and cross-sectional scanning electron microscopy (SEM) imaging (Nova Nano 450, ThermoFisher, Waltham, MA, USA), respectively, while the types and relative amounts of the constituting phases were determined by X-ray diffraction (XRD, D2 Phaser, Bruker Co., Billerica, MA, USA). The porosity of each film was determined by initially measuring its mass before and after deposition using a high-precision electronic scale (EX225/AD, OHAUS Co., Parsippany, NJ, USA) with a resolution of up to 10^−5^ g and then comparing the obtained values with the bulk density of zinc oxide (5.61 g/cm^3^). The density of porous ZnO films could be calculated by dividing the measured mass of the films by the product of the deposition area and the average thickness of the films. The porosity of ZnO films could be obtained by using Equation (1).

(1) $$\mathrm{Porosity}\;\mathrm{of}\;\mathrm{ZnO}\;\mathrm{film}\left(\%\right)=\frac{\mathrm{Bulk}\;\mathrm{density}\;\mathrm{of}\;\mathrm{ZnO}-\mathrm{Density}\;\mathrm{of}\;\mathrm{ZnO}\;\mathrm{film}}{\mathrm{Bulk}\;\mathrm{density}\;\mathrm{of}\;\mathrm{ZnO}}\times 100$$

## 3. Results and Discussion

**Figure 2:**
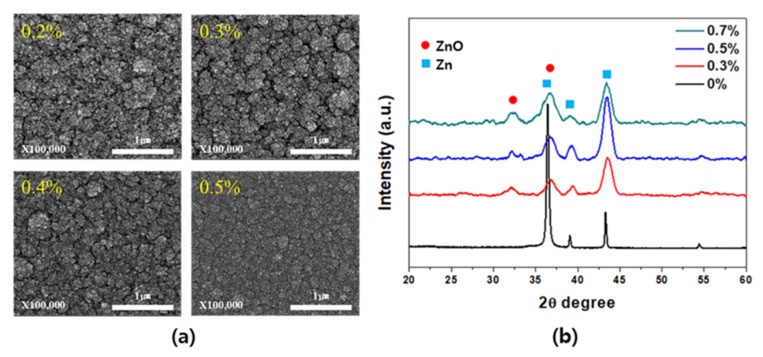
(**a**) Surface SEM images and (**b**) XRD patterns of the formed Zn and ZnO films upon variation in the O_2_ ratio.

Figure 2 shows the changes in the surface SEM images and XRD patterns of the porous Zn and Zn-ZnO composite films with variation in the O_2_ ratio relative to Ar. The pure porous Zn film fabricated by injecting only Ar gas into the system exhibited a porosity of about 40% but showed poor substrate adhesion and low reliability. Following the addition of O_2_ gas to the Ar stream, porous Zn-ZnO composite films were obtained that exhibited improved adhesion properties and a superior reliability while maintaining the porosity of the original Zn film. Figure 2a shows the changes in the particle size and distribution as a function of the O_2_ ratio. As the O_2_ ratio was increased, the particle size increased, and the porosity decreased, indicating a tendency of increasing density. The XRD results presented in Figure 2b show a strong Zn peak when Ar gas alone was employed, whereas both Zn and ZnO were present when the O_2_ ratio was ≥0.3%. However, at an O_2_ ratio of ≥0.5%, dense films were obtained, and so a ratio of 0.3% was selected to maintain the optimal porosity. Compared to the pure porous Zn films, the Zn-ZnO composite films were thought to have relatively superior adhesion to the substrate through the results of 3M tape, scratch, and gas injection tests.

**Figure 3:**
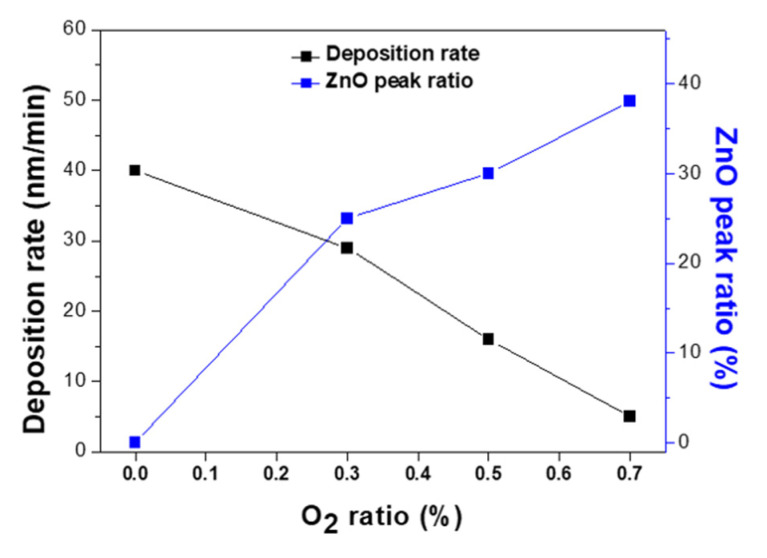
Deposition rates and ZnO peak ratios of the films upon variation in the O_2_ ratio.

Figure 3 shows the variations in the film deposition rate and ZnO peak ratio upon altering the O_2_ ratio relative to Ar. The ZnO peak ratio could be obtained by using Equation (2). In the equation, the ZnO peak area is obtained by integrating the region around ZnO (100) peak in the XRD pattern. Similarly, the Zn peak area is the value calculated from the Zn (101) XRD peak.

(2) $$\mathrm{ZnO}\;\mathrm{peak}\;\mathrm{ratio}\left(\%\right)=\frac{\mathrm{ZnO}\;\mathrm{peak}\;\mathrm{area}}{\mathrm{ZnO}\;\mathrm{peak}\;\mathrm{area}+\mathrm{Zn}\;\mathrm{peak}\;\mathrm{area}}$$

More specifically, in an oxygen-free atmosphere, the deposition rate was 40 nm/min, while at O_2_ ratios of 0.3% and 0.7%, the rate dropped to 29 and 5 nm/min, respectively. This reduction in the deposition rate with increasing O_2_ ratios was attributed to the increased number of ZnO particles suppressing the formation of Zn clusters. In addition, due to the fact that the ZnO particles were expected to have a higher mobility on the substrate surface compared to the Zn clusters, the film porosity decreased at higher O_2_ ratios. Furthermore, the film with a higher ZnO peak area ratio was obtained at a higher O_2_ ratio as zinc and oxygen could be easily combined.

**Figure 4:**
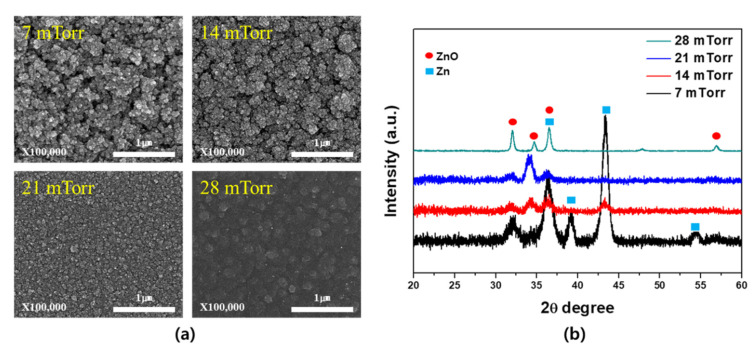
(**a**) Surface SEM images and (**b**) XRD patterns of the formed Zn and ZnO films upon variation in the process pressure.

Figure 4 shows the surface SEM images and XRD patterns obtained upon variation in the process pressure at the optimized O_2_ ratio of 0.3%. More specifically, Figure 4a illustrates the changes in the particle size and distribution at each pressure. Based on these observations, it appeared that the porosity decreased as the process pressure increased, and this was attributed to the fact that contact with oxygen became more likely at higher pressures, thereby promoting the formation of the highly mobile ZnO on the substrate surface and increasing the film density. As shown in the XRD measurement results in Figure 4b, a film containing both Zn and ZnO was formed even at a deposition pressure of 7 mTorr, although the peak intensity of the Zn component was significantly higher. As the pressure was increased, the peak intensity of the Zn component decreased, whereas that corresponding to ZnO increased, thereby implying an increased amount of ZnO formation. This observation indicates that higher process pressures generate greater quantities of gas in the plasma, thereby increasing the probability of interactions between Zn and O_2_ and resulting in a greater amount of ZnO.

**Figure 5:**
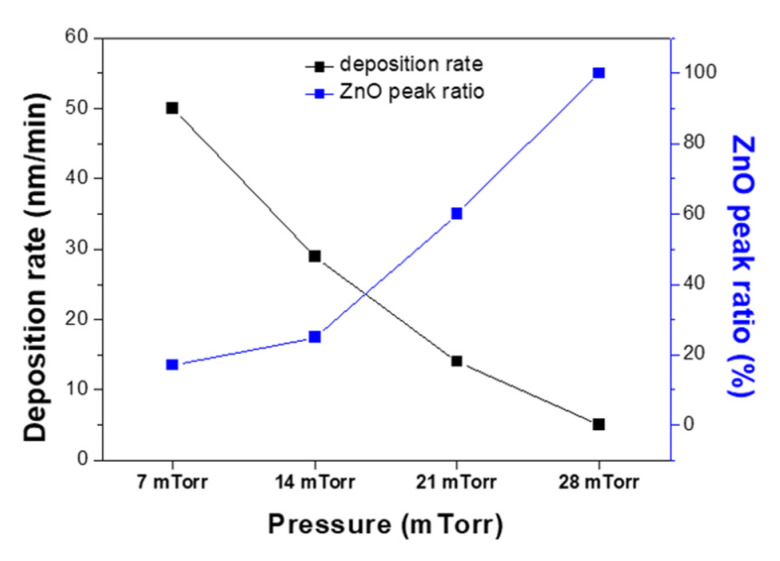
Deposition rates and ZnO peak ratios of the films upon variation in the process pressure.

Figure 5 illustrates the change in the deposition rate and ZnO peak ratio upon variation in the process pressure at a constant O_2_ ratio of 0.3%. As indicated, the deposition rate was lower at higher pressures, likely because the number of collisions between particles increased, which reduced the kinetic energy of particles and, in turn, the number of particles reaching the substrate. Densification caused by the increased formation of ZnO particles under high pressures was also considered to play a role in this decrease in the deposition rate. The ZnO peak ratio increased with the increase in pressure. The ratio reached almost 100% at a pressure of 28 mTorr. The increase in the collision between particles could enhance the bonding of zinc and oxygen, which have higher energy.

**Figure 6:**
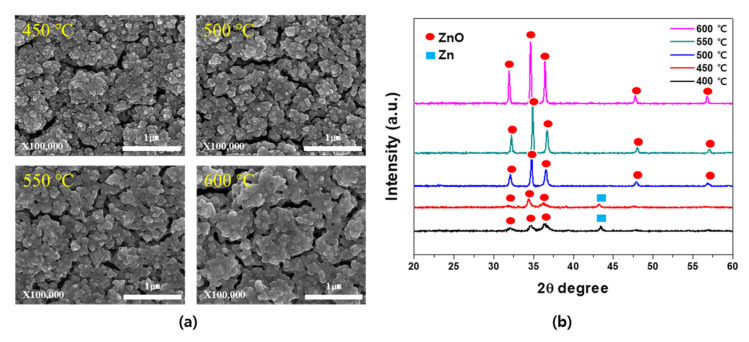
(**a**) Surface SEM images and (**b**) XRD patterns of the formed Zn-ZnO films upon variation in the oxidation temperature.

Figure 6 shows the SEM images and XRD patterns of the films fabricated through the wet post-oxidation treatment of the porous Zn-ZnO composite films at various oxidation temperatures, which were prepared at an O_2_ ratio of 0.3% and a process pressure of 14 mTorr. More specifically, Figure 6a shows the changes in the particle size and distribution upon variation in the oxidation temperature. As indicated, similar results were obtained between 450 and 500 °C, but the particle size started to increase with decreasing porosity at 550 °C, while at 600 °C, the particle size increased significantly. Figure 6b shows the corresponding XRD results, which indicate the presence of a Zn peak at temperatures up to 450 °C and the presence of a peak corresponding to the pure porous ZnO at 500 °C. As the oxidation temperature was increased, the intensity of the ZnO peak also increased. This was attributed to the fact that oxidation treatment at a high temperature weakened the bonding in the Zn clusters and facilitated the reaction with the incoming water vapor to form ZnO, resulting in overall oxidation [31,32]. For the oxidation reaction to proceed to a point where no residual Zn was present, an oxidation temperature of >500 °C was required. However, for the purpose of this study, an oxidation temperature of 500 °C was selected to minimize the decrease in porosity caused by the coarsening of particles with increasing temperature.

**Figure 7:**
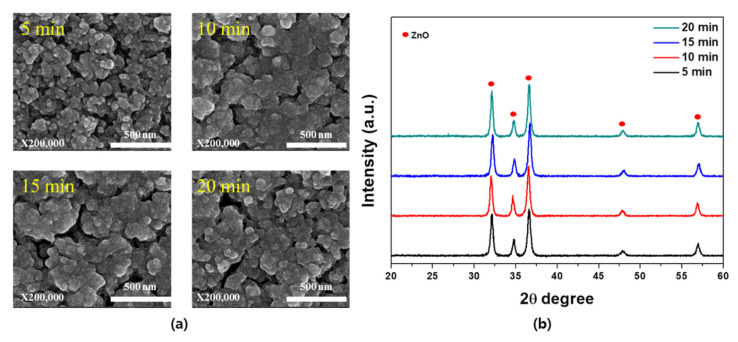
(**a**) Surface SEM images and (**b**) XRD patterns of the formed ZnO films upon variation in the oxidation time.

Figure 7 shows the SEM images and XRD patterns of the optimized Zn-ZnO composite films with respect to the oxidation time under a wet post-oxidation temperature of 500 °C. More specifically, Figure 7a shows the changes in the particle size and distribution upon variation in the oxidation time, wherein it can be seen that the grain size increased due to the merging of adjacent grains over time. Based on the XRD results presented in Figure 7b, an oxidation time of 5 min was considered sufficient to obtain a pure porous ZnO film, since a shorter time led to highly porous ZnO films.

**Figure 8:**
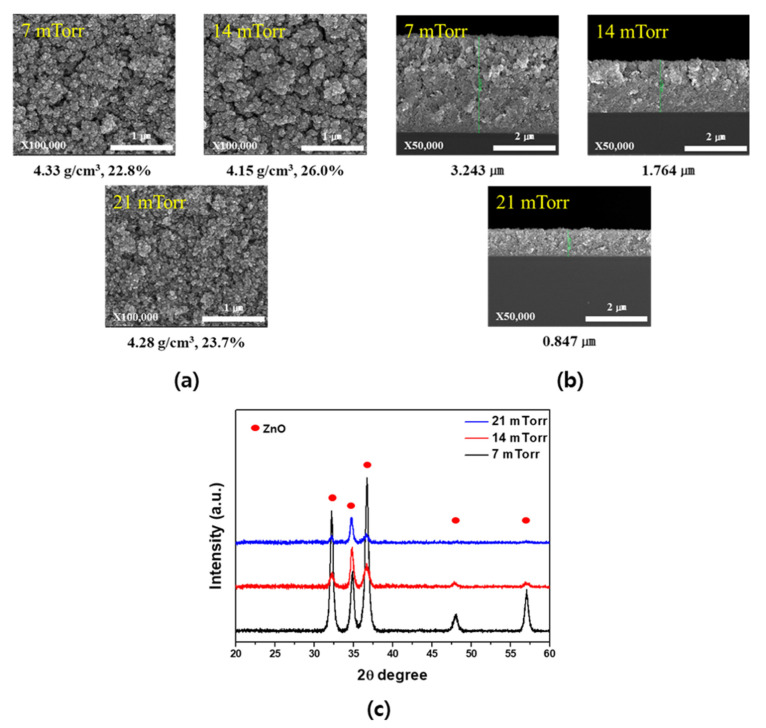
(**a**) Surface SEM images (corresponding densities and porosities indicated below the images), (**b**) cross-section SEM images (corresponding thicknesses indicated below the images), and (**c**) XRD patterns following the wet post-oxidation process at various deposition pressures.

Based on the above results, the optimized conditions for the wet post-oxidation process were taken as an oxidation temperature of 500 °C and an oxidation time of 5 min. Figure 8 shows the changes in the SEM images and XRD patterns with variation in the deposition pressure following the optimized wet post-oxidation process for the Zn-ZnO composite films. More specifically, Figure 8a shows the surfaces of the obtained films, wherein upon comparison with the images shown in Figure 4a, no significant differences in the surface shape were observed, and the pore structure was almost maintained. The density and porosity were also calculated and were found to range from 4.15 to 4.33 g/cm^3^ and from 23% to 26%, respectively. The cross-section SEM images are shown in Figure 8b, in which the observed thicknesses were used to calculate the density and porosity values in Figure 8a. Furthermore, Figure 8c shows the XRD patterns obtained at the various process pressures, wherein it is apparent that under all conditions examined, a pure porous ZnO film was obtained. Based on these results, we fabricated Zn-ZnO composite films under a process pressure of 14 mTorr and an O_2_ ratio of 0.3%, followed by wet oxidation at 500 °C for 5 min to obtain ideal porous ZnO films.

**Figure 9:**
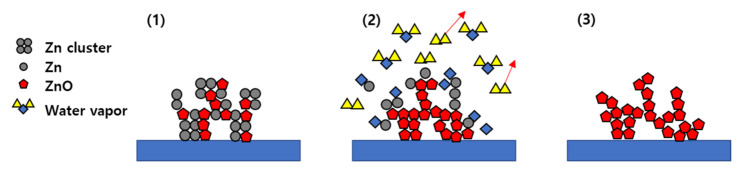
Mechanism of oxidation for the porous Zn-ZnO composite films. (**1**) Deposition of Zn-ZnO composite cluster, (**2**) Reaction of water vapor with Zn in the cluster, and (**3**) Formation of highly porous ZnO film.

Finally, Figure 9 shows the mechanism by which a porous Zn-ZnO composite film is oxidized to yield a pure porous ZnO film. More specifically, in stage 1, the Zn and ZnO clusters are deposited together to form a porous structure. In stage 2, the wet post-oxidation process is conducted at 500 °C, which is higher than the melting point of Zn (i.e., 420 °C), and so the Zn present in the clusters melts, thereby weakening the bonding and reacting with the introduced water vapor to form ZnO. However, at a lower temperature, Zn remains in the film as a result of an insufficient reaction with water. In stage 3, the prepared ZnO acts as a support during the oxidation process, which allows the porosity to be maintained while forming pure porous ZnO films. However, higher oxidation temperatures or longer oxidation times result in grain growth due to the transport of ZnO particles, which reduces the porosity and gives a denser structure.

## 4. Conclusions

The formation of porous Zn-ZnO composite films was examined using the modified direct current (DC) magnetron sputtering method, followed by the formation of pure porous ZnO films through subsequent heat treatment in the presence of water vapor. Upon variation in the O_2_ content of the Ar gas flow during Zn-ZnO film deposition, it was found that an O_2_ ratio of 0.3% resulted in the best porosity, with any further increase in the O_2_ content leading to a denser surface on the obtained Zn-ZnO film. In addition, upon variation in the process pressure, a pressure of 14 mTorr was found to be optimal. Although the deposition rate was higher at a lower process pressure, the probability of reaction was reduced, which led to films containing mainly Zn, and only small amounts of ZnO were detected. At a higher process pressure, the particles lost significant amounts of energy through more frequent collisions, thereby reducing the amount of deposition and increasing the amount of ZnO to give a film with low porosity. Furthermore, at a low oxidation temperature, an X-ray diffraction peak corresponding to residual Zn was observed, while upon increasing the oxidation temperature to ≥500 °C, pure porous ZnO films were obtained. However, it should be noted that the film density also increased at higher temperatures. Upon varying the oxidation time, the porosity decreased as the particles grew over time; a pure ZnO film was formed within 5 min. Ultimately, we were able to obtain a porous ZnO film with a porosity of 26% by depositing a Zn-ZnO composite film at a process pressure of 14 mTorr and an O_2_ ratio of 0.3%, followed by oxidation of the film at 500 °C for 5 min. Due to the fact that the films produced using this method are highly reliable, we expect that they could be suitable for applications that require large specific surface areas, such as sensors, supercapacitors, and batteries.

## Figures and Tables

**Table 1:** Summary of the experimental parameters used in this study for fabrication of the porous zinc oxide thin films.

Deposition	Variable Parameters	Operating pressure (mTorr)	7–28
		Gas ratio (O_2_, %)	0–0.7
	Fixed Parameters	Chiller temperature (°C)	20
		Deposition time (min)	60
		Power (W)	150
		Argon flow rate (sccm)	10
Oxidation	Variable Parameters	Oxidation temperature (°C)	400–600
		Oxidation time (min)	5–30
	Fixed Parameters	Gas species	N_2_ + H_2_O
		Carrier gas flow rate (LPM)	0.7

## Data Availability

The data presented in this study are contained within the article.
